# Development and Feasibility of a Smartphone, ECG and GPS Based System for Remotely Monitoring Exercise in Cardiac Rehabilitation

**DOI:** 10.1371/journal.pone.0014669

**Published:** 2011-02-09

**Authors:** Charles Worringham, Amanda Rojek, Ian Stewart

**Affiliations:** Institute of Health and Biomedical Innovation, Queensland University of Technology, Brisbane, Queensland, Australia; Universidad Peruana Cayetano Heredia, Peru

## Abstract

**Background:**

Despite its efficacy and cost-effectiveness, exercise-based cardiac rehabilitation is undertaken by less than one-third of clinically eligible cardiac patients in every country for which data is available. Reasons for non-participation include the unavailability of hospital-based rehabilitation programs, or excessive travel time and distance. For this reason, there have been calls for the development of more flexible alternatives.

**Methodology and Principal Findings:**

We developed a system to enable walking-based cardiac rehabilitation in which the patient's single-lead ECG, heart rate, GPS-based speed and location are transmitted by a programmed smartphone to a secure server for real-time monitoring by a qualified exercise scientist. The feasibility of this approach was evaluated in 134 remotely-monitored exercise assessment and exercise sessions in cardiac patients unable to undertake hospital-based rehabilitation. Completion rates, rates of technical problems, detection of ECG changes, pre- and post-intervention six minute walk test (6 MWT), cardiac depression and Quality of Life (QOL) were key measures. The system was rated as easy and quick to use. It allowed participants to complete six weeks of exercise-based rehabilitation near their homes, worksites, or when travelling. The majority of sessions were completed without any technical problems, although periodic signal loss in areas of poor coverage was an occasional limitation. Several exercise and post-exercise ECG changes were detected. Participants showed improvements comparable to those reported for hospital-based programs, walking significantly further on the post-intervention 6 MWT, 637 m (95% CI: 565–726), than on the pre-test, 524 m (95% CI: 420–655), and reporting significantly reduced levels of cardiac depression and significantly improved physical health-related QOL.

**Conclusions and Significance:**

The system provided a feasible and very flexible alternative form of supervised cardiac rehabilitation for those unable to access hospital-based programs, with the potential to address a well-recognised deficiency in health care provision in many countries. Future research should assess its longer-term efficacy, cost-effectiveness and safety in larger samples representing the spectrum of cardiac morbidity and severity.

## Introduction

Cardiac rehabilitation (CR) is widely recognised as playing a critical role in optimising recovery in cardiac patients, with meta-analyses demonstrating reduced cardiac and all-cause mortality, fewer cardiovascular related events, less re-hospitalisation and shorter length of stay. [1]-[3] CR has also been shown to be a highly cost effective form of secondary prevention. [2], [4]

Unfortunately, limited accessibility and low participation levels present persistent challenges in almost all countries where CR is available. In Australia as few as one in five of those who are clinically eligible actually complete CR, [5], [6] with a figure of approximately one in three in the United Kingdom (where the National Service Framework has set a target of 85% invitations to participate), and comparably low figures have been reported for the United States. [7] A spate of recent studies have characterised the factors that determine non-participation. While poor referral practices mitigate against participation, [8], [9] and older patients [8], females [8], or those with more clinical complications [8], [9], [10] participate in or complete programs at lower rates, there are also more immediate access barriers which have been consistently reported. These include living further from or taking longer to travel to a program, [8], [9] or being unable to drive. [10]

Despite this improved understanding of barriers to participation, studies reporting possible *solutions* have been much less numerous. This is despite strong calls for the exploration of more flexible CR programs [11], including the observation that *“the potential for embracing novel methods and the latest technology are rarely exploited”* [12] and the fact that those living in rural and remote locations with the least access to conventional CR have higher levels of cardiac morbidity and mortality than those in metropolitan areas. [13]

Home-based CR offers a potentially valuable alternative for such individuals, and in one RCT showed comparable improvements to a centre-based program across a range of measures, [14] at lower [15] or comparable cost [16], [17] However, these programs vary widely in mode of delivery, content and supervision, and may have minimal or no monitoring of risk factors and heart function. The lack of functional monitoring may not only amplify safety concerns, it can also limit the ability to rapidly modify clinical management. In one study of 3,877 hospital-based CR exercise sessions, for example, 24% of low-risk and 34% of medium and high-risk patients showed an “untoward event”, of which 56% were initially detected with ECG, more than half of which occurred in the first two weeks, and 58% of which resulted in a change to the patient's medical management. [18]

Home-based rehabilitation should ideally permit functional monitoring, and one such method, trans-telephonic transmission of baseline or exercise ECG, which was first trialled twenty-five years ago [19] has had generally positive evaluations, [20]-[22]. A disadvantage is that available systems restrict users to a land-line and thus to exercising indoors in a fixed location. There is a need for more flexible, cost-effective and customised forms of exercise rehabilitation which can be conducted at any suitable location.

We report here the development of a system to meet this need, together with an initial evaluation of its outcomes and feasibility. Patients undertook outdoor walking exercise at pre-arranged times, either near their home, work, when visiting friends or on holiday breaks or while travelling. Outdoor walking is particularly suitable for CR not only because it can impose an appropriate and readily modifiable workload and is generally seen as enjoyable, but because it has a crucial function in the resumption of work and daily life. During the exercise sessions reported here, the patient's location, speed, heart rate and single lead ECG were remotely monitored in real-time by a university-trained exercise scientist.

## Materials and Methods

### Study Population

**Figure 1:**
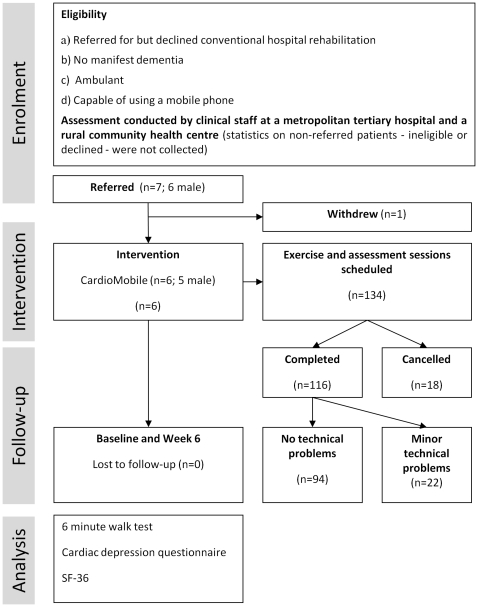
Flowchart of trial methodology.

Exercise sessions were conducted in a small series of cardiac patients who were eligible for, but unable to attend conventional CR. Participants had been discharged from hospital following a coronary event and/or coronary revascularisation surgery (percutaneous transluminal coronary angioplasty or coronary artery bypass grafting). Referrals of eligible patients were made by clinical staff at a metropolitan tertiary private hospital and a rural community health centre. Of the first seven referred patients, six (one female and six male) completed the study and one (male) withdrew prior to initial exercise testing. This small sample was sufficient to allow in excess of 100 monitored exercise sessions to be undertaken as a basis for the feasibility assessment. The recruitment pathway and trial methodology are depicted in Figure 1. Ethical approval was obtained for this study from the Queensland University of Technology Human Research Ethics Committee and all participants provided informed consent.

### Procedures and Exercise Program

Patients completed an average of three outdoor walking sessions per week for six weeks. Exercise duration and intensity was individually prescribed following a modified 6-minute walk test (6 MWT) using GPS conducted in separate remotely-monitored sessions before and after the exercise intervention, and using GPS data to measure elapsed distance. Programs were periodically revised on the basis of performance and symptoms, by increasing distance, incorporating hills, varying pace, and for two participants where deemed appropriate, short periods of jogging.

At the time scheduled for a session, the participant applied the electrodes and turned on the equipment. As soon as signals were received at the base station the patient was phoned, ECG signal quality checked, and the goals for that session confirmed. The testing protocol required that if during any session cardiac symptoms were reported, or if there was an unexplained interruption of data, or if clinically significant ECG changes were detected, the patient would be contacted by phone and follow-up measures taken (exercise cessation or modification, or consultation with the patient's doctor). The location and status of the patient could be conveyed immediately to local emergency services if needed.

### Development of the Remote Monitoring System

**Figure 2:**
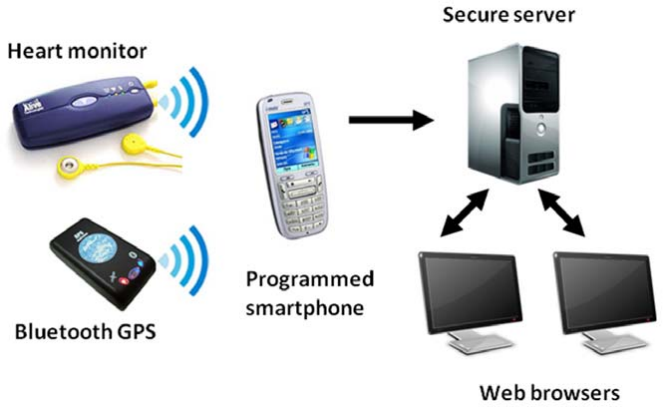
Components of remote monitoring system.

**Figure 3:**
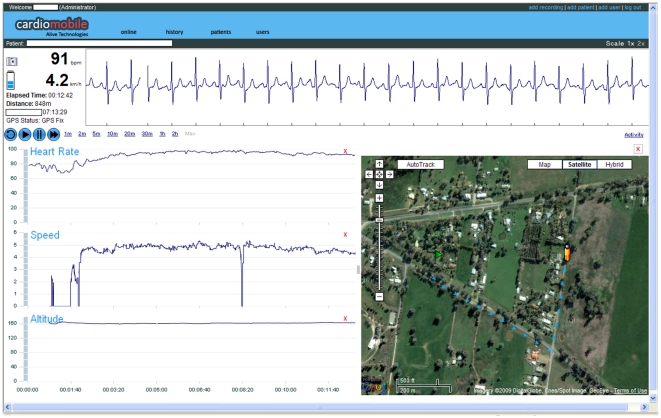
Screen-shot of server-side display of exercise data.

The remote monitoring system developed in this project comprised a miniature heart and activity monitor worn in a pouch on the belt, recording single lead ECG at 300 Hz (Alive Technologies Pty Ltd, Australia), a miniature non-differential 1 Hz GPS receiver (Wonde Proud, Taiwan) worn in a small pouch at the back of a cap, and a programmed smartphone (i-Mate SP3, Dubai) (Figure 2). The monitor and GPS receiver communicated with the smartphone via Bluetooth®. The set-up and programming of these devices was designed to minimise demands on users who may have no previous experience with mobile technology. In particular, no dialling was required and users had only to turn on the devices and turn them off after use. The smartphone keypad was locked to prevent accidental activation. During exercise sessions, the smartphone streamed GPRS data to a secure server enabling the ECG trace, heart rate, walking speed, elapsed distance and patient location to be viewed in real-time (Figure 3). Server-side software was developed to allow the display of incoming signals and system status, with a sweep-display of the ECG signal, self-scaling displays of heart rate and walking speed, elapsed time and distance indicators, and a map or satellite image display of the patient's location and path. Participants were provided with voice only mobile phone for pre- and post-session contact with the exercise scientist, and for routine or emergency contact during a session if needed.

### Outcome Measures

In addition to the assessment of exercise capacity with the 6 MWT, patients completed the Cardiac Depression Questionnaire [23] and a Generic Health Related Quality of Life Questionnaire Short Form-36 (SF-36) before the intervention, and again after, when they also rated 14 aspects of the equipment's useability, and the frequency of six different possible technical problems. They also estimated the time taken to set up and take off the equipment, and provided qualitative statements about their experience of the program. Pre-post comparisons of these measures were made with paired t-tests.

## Results

The six participants had an average age of 53.6 (42–67) years, an average BMI of 25.9 (19.6–33.4), and cited work commitments, distance to hospital, work-related travel, or lack of transport as reasons for declining hospital-based CR. All participants who commenced exercise sessions completed a customised six-week rehabilitation program, with a total of 134 walking-based exercise or exercise assessment sessions being scheduled, 116 completed (86.6%) and 18 cancelled (13.4%) (Table 1). At no time were symptoms reported or ECG signs observed (e.g. ventricular trachycardia/fibrillation) that required emergency management. There were two occasions on which asymptomatic ECG changes prompted the research team to consult the patient's cardiologist before resuming exercise sessions. These included ST segment changes as well as exercise and post-exercise supraventricular and ventricular ectopic beats.

**Table 1:** Session status and technical problems.

Completion status	*n*	*%*	Session status	*n*	*%*
Completed	116	86.6	No technical problems	94	70.1
			Intermittent signal loss<15 s	10	7.5
			Battery ran out towards end	6	4.5
			Other intermittent technical problems	2	1.5
			Poor ECG signal	4	3.0
Cancelled	18	13.4	Bad weather	6	4.5
			Other technical problems	4	3.0
			Patient unavailable	3	2.2
			Inadequate phone coverage	2	1.5
			Battery not recharged	2	1.5
			Poor ECG signal	1	0.7
Total	134	100.0		134	100.0

Over 80% of completed sessions had no technical problems. Of the remaining sessions, intermittent signal interruption attributable to poor mobile phone coverage was the most common problem, followed by loss of battery power (Table 1).

Participants rated the usability of the system favourably (Ease of use rating: mean, 4.8; 95% CI, 4.6–5.0, where 4 = quite easy and 5 = very easy; Frequency of technical problems rating: mean, 4.; 95% CI, 4.1–4.9, where 4 = less than once a week and 5 = never), reporting an average of 3.9 minutes to put on and 3.0 minutes to remove the equipment following a session.

Improvements in physical and psychological function on completion of the program are presented in Table 2. Patients showed statistically significant improvements in walking distance, cardiac depression, and the physical (but not the mental) health component of the SF36 Health Related Quality of Life questionnaire. Distance walked on the 6 MWT increased by an average of 113 m, comparable to published reference values [24] for the effects of cardiac rehabilitation on the 6 MWT.

**Table 2:** Functional outcome measures.

	Pre-Intervention		Post-Intervention		*P* (Pre vs. Post)*
	Mean	95% CI	Mean	95% CI	
Six Minute Walk test (m)	524	420–655	637	565–726	0.009
Cardiac Depression	54.0	40.4–71.0	44.6	32.0–60.4	0.007
SF36 (Physical health)	50.0	36.0–67.5	78.4	57.9–103.9	0.03
SF36 (Mental health)	67.4	44.4–96.3	71.5	54.6–92.8	0.5 (n.s.)

*Two-tailed dependent scores t-test.

## Discussion

This study shows that outdoor, walking-based cardiac rehabilitation with real-time remote monitoring of ECG, HR, location and speed is technically feasible and can be used to provide supervised CR to patients unable to access hospital-based programs.

From a technical and ease-of-use perspective, participants could use the equipment readily and quickly. Since target populations include those who may be less familiar with portable electronic devices, simple operation is essential, and restricting the operational tasks to turning the devices on and off simplified these human factors demands. Although the majority of sessions had no technical problems there was periodic signal interruption, mostly in rural areas, because of limitations in GPRS coverage. One participant was travelling between the states of Queensland and Victoria during his rehabilitation and often exercised at new locations that could not be checked in advance for adequacy of coverage. These coverage limitations have subsequently been greatly reduced by using Australia's 850 mHz 3G network, which provides greater bandwidth, more robust and much more widespread connectivity, particularly in rural and remote areas. Forthcoming wireless network innovations such as Time Division Multiple Access (TDMA) will only further optimize performance. Less often, a participant had forgotten to recharge the phone battery, so reminders were added to the session protocols after exercise. ECG signal quality was generally good and enabled the identification of ST segment changes, supraventricular and ventricular ectopic beats, but on occasion it was adequate or very poor. This was usually improved by asking the participant to adjust the electrode leads to prevent tugging and consequent movement artifacts, or to prepare the skin again and replace the electrodes.

Conventional cardiac rehabilitation has proven very safe, with one event every 49,565 patient-hours of exercise training and one cardiac arrest for every 1.3 per million patient-hours of exercise. [25] Patients undertaking home-based CR are not necessarily at greater risk of adverse events, but because emergency services are not immediately available, monitoring and supervision of the type provided in this study can be beneficial in two distinct ways. Two of the six participants showed ECG changes that warranted medical consultation, although both were cleared to complete the program. The potential detection of more serious abnormalities, together with voice contact and GPS location offer a real degree of improved safety compared to a patient exercising with no supervision or monitoring. Nevertheless, future research should give specific consideration to patient safety, which can present special challenges when rehabilitation is conducted at a distance from emergency care. For some high risk patients, or in potentially hazardous locations (such as those with traffic hazards, unsafe walking surfaces or areas with inadequate public safety), this approach will need careful assessment of safety risks as well as health benefits.

Of equal or possibly greater benefit is the psychological reassurance such monitoring can provide in the critical early weeks of recovery. A recent UK study of cardiac patients noted that a common belief amongst patients was that exercise rehabilitation was potentially harmful. [26] In the current study, participants reported that being monitored allowed them to feel more confident about exercising than would otherwise have been the case, and that the level and intensity of exercise was appropriate. It is likely that participation, compliance and outcomes benefit from this reassurance. While ECG monitoring may therefore have motivational as well as clinical value, our view is that it should be phased out after the initial weeks to prevent dependency. For example, a study of hospital-based CR showed that patients whose ECG monitoring was discontinued after one month undertook more off-site exercise and had higher exercise self-efficacy over the six-month follow-up than those who were monitored throughout rehabilitation. [27]

The study also successfully employed GPS recording to undertake six minute walk tests remotely. This approach has been previously validated for determining maximal walking distance in a population with Peripheral Arterial Disease.[28] The system used in this study is highly accurate for determining velocity across all human walking and running speeds, [29] which allows for precise calculation of physiological work undertaken. Accurate location data allows changes in physiological parameters (heart rate) to be related to changes in the external environment (e.g. walking up steps or hills) and enables exercise programs to be tailored to suit individual capacity and progression.

### Limitations

The study had two principal limitations. Although the improvements noted in walking distance, cardiac depression and physical health QOL are comparable to those observed in hospital-based CR, they cannot be unambiguously attributed to the intervention because, as a feasibility trial, no control group was required and therefore no comparisons with alternative interventions were possible. A second limitation was the small and predominantly male study group. Despite these limitations, we submit that the number of successfully completed and monitored sessions (116), the acceptance of the technology, the high compliance rate and the promising outcomes are grounds for the conduct of additional studies. These should further consider safety and efficacy, and extend the research beyond technological considerations to larger-scale and longer-term evaluations of the clinical, functional and quality of life benefits to the patient, as well as cost-benefit analyses. Comparisons with usual care (which, for the target population of those without access to programs is *no* formal rehabilitation), as well as with conventional treatment (hospital-based rehabilitation) would be important. If this approach proves safe, beneficial and cost-effective, its adoption could help to address a well-recognised deficiency in health care provision (in many countries), and which is particularly marked for patients in rural and remote areas.
